# Dilution as a Solution: Targeting Microbial Populations with a Simplified Dilution Strategy

**DOI:** 10.1128/mSphere.00701-20

**Published:** 2020-07-29

**Authors:** Disha Bhattacharjee, Anna M. Seekatz

**Affiliations:** a Department of Biological Sciences, Clemson University, Clemson, South Carolina, USA

**Keywords:** *C. difficile*, FMT, microbiota, minibioreactors

## Abstract

The gut microbiota is an integral part of maintaining resistance against infection by *Clostridioides* (*Clostridium*) *difficile*, a pathogen of increasing concern in both health care and community settings. The recent article by J. M. Auchtung, E. C. Preisner, J. Collins, A. I. Lerma, and R. A. Britton (mSphere 5:e00387-20, 2020, https://doi.org/10.1128/mSphere.00387-20) demonstrates an innovative approach to identify microbes that inhibit C. difficile by employing a dilution scheme to test different microbial mixtures *in vitro* and *in vivo*. This type of approach can advance the identification and validation of specific microbes that elicit functions of interest for many conditions involving the microbiota, of which the complexity and variability can often complicate causality.

## Abstract (precis)

The gut microbiota is an integral part of maintaining resistance against infection by *Clostridioides* (*Clostridium*) *difficile*, a pathogen of increasing concern in both health care and community settings. The recent article by J. M. Auchtung, E. C. Preisner, J. Collins, A. I. Lerma, and R. A. Britton (mSphere 5:e00387-20, 2020, https://doi.org/10.1128/mSphere.00387-20) demonstrates an innovative approach to identify microbes that inhibit C. difficile by employing a dilution scheme to test different microbial mixtures *in vitro* and *in vivo*.

## COMMENTARY

The development of microbial therapeutics to target infections and other health conditions has surged in the last decade. One microbial application, fecal microbiota transplantation (FMT), has received particular attention due to its high rate of success in treating *Clostridioides* (*Clostridium*) *difficile* infection (CDI), an important health care-acquired infection. Because the use of undefined concoctions of fecal material may contain harmful bacterial, viral, fungal, and other components that influence health downstream, strategies to identify a targeted microbial population to treat CDI are of interest. However, both inherent variability in successfully used microbial communities and the ability to rapidly screen these diverse microbes have hindered these efforts. A recent study by Auchtung et al. developed an experimental platform to define bacterial components in the human microbiota that may elicit protection against C. difficile, combining *in vitro* and *in vivo* approaches to simplify these complex communities (1). Although that study focuses on CDI, the same approach can be applied to other gastrointestinal diseases influenced by the gut microbiota.

Auchtung et al. first applied a dilution scheme to six fecal samples collected from healthy human participants to generate multiple simplified microbial communities. Multiple diluted communities (at 10^−4^ and 10^−5^ dilutions) were individually seeded into minibioreactor arrays, an approach that has been previously used to study microbiota functions (2). Minibioreactor cultures were then tested for the ability to inhibit C. difficile spores. Of these, approximately a third of the simplified communities were able to inhibit C. difficile growth by at least 10^4^. The use of 16S rRNA gene-based sequencing revealed that dilution decreased the total number of operational taxonomic units (OTUs) in the simplified communities resistant to C. difficile from 62 to 42, with each dilution composed of taxonomically diverse microbes that approximated their donor origin. The investigators then chose a subset of microbial mixtures to test their abilities to inhibit C. difficile in a mouse model of disease. Mice with an established “humanized” microbiota (3) were pretreated with antibiotics, followed by gavage with one of five simplified, resistant communities and C. difficile challenge. Two of the five communities tested demonstrated rapid clearance of C. difficile, both following initial challenge and later with relapse. Notably, mice that cleared C. difficile also maintained higher body weight than mice that continued to shed C. difficile, indicating protection from disease. One of the selected resistant communities that was further diluted (to 28 and 9 OTUs) continued to restrict C. difficile expansion. Interestingly, the majority of the organisms in these samples were classified as *Clostridiales*. However, microbial recovery in animals with CDI that were treated with simplified communities was not restricted to organisms found in the input; in fact, OTU tracking suggested that OTUs present in the mouse prior to antibiotic treatment were also significantly enriched. These included OTUs belonging to multiple phyla, such as *Bacteroides*, *Parabacteroides*, E*rysipelotrichaceae*, *Bifidobacterium*, and *Porphyromonadaceae* OTUs.

These data suggest two important points concerning the identification of strains that could ultimately provide support for targeted microbial treatments against CDI: (i) a diverse set of microbes is likely able to inhibit C. difficile and (ii) microbes introduced to the system are only part of the recovery process. Much of the focus of CDI therapeutics has focused on identifying microbes that provide functions known to target C. difficile itself, such as the production of secondary bile acids that inhibit C. difficile growth (4). Other groups have focused on groups of bacteria previously associated with recovery of the microbiota, such as spore-forming bacteria, which include *Clostridiales* (5, 6). Yet data suggest that defining the magic bullet against C. difficile is perhaps not as stringent. Previous studies have demonstrated that communities of microbes without the obvious capability to produce secondary bile acids are sufficient to clear C. difficile in mice (7). Notably, the simplified communities identified as inhibitory to C. difficile did not necessarily harbor any one group of microbes but contained a spectrum of organisms. Instead, as demonstrated in the mice that recovered from CDI following successful microbial intervention, the microbial input may serve as a mechanism to induce expansion of extant host microbiota that eventually inhibit C. difficile. This has been demonstrated to some extent in human FMT studies (8, 9). How the host and its microbes “accept” these microbes may be key to understanding how to modulate this environment.

Cultivation of microbes of interest remains an obstacle to development of targeted microbial therapeutics, for CDI and otherwise. The methodology employed by the authors provides a potential solution to isolation, screening, and validation of complex microbial communities, which are often laborious. While this study is not the first to use a dilution/extinction strategy to simplify communities (10) or bioreactors (11), the combination of *in vitro* and *in vivo* approaches to screen simplified communities for a particular output is efficient and easily adapted for other uses. *In vitro* prescreening of defined microbiota mixtures reduces the number of animals necessary for testing multiple combinations of microbes. The approach is also highly applicable for the identification of microbial communities important in other conditions where the microbiota is assumed to be involved. Minibioreactors for identification, screening, and validation of defined microbial populations could easily be modified to look for specific output, such as production of specific metabolites under different conditions or the impact of environmental disruptions on the community function. Indeed, a logical inclusion to this study would have been to use the bioreactor system to identify whether metabolites known to impact C. difficile were produced by the simplified communities. This additional measurement may have identified shared functions of seemingly diverse microbial mixtures that influence C. difficile physiology, as well as provide some context for how well these functions are recapitulated *in vivo*.

In summary, Auchtung et al. demonstrate a novel approach to identify distinct microbes of interest. As interest in the development of microbial therapeutics to treat disease and maintain health continues, efficient systems as described in that study can advance the process of understanding these complex microbial communities.
